# Association Between Asthma and Migraine: A Systematic Review and Meta-Analysis of Observational Studies

**DOI:** 10.3389/falgy.2021.741135

**Published:** 2021-12-01

**Authors:** Lin-Lin Kang, Pei-En Chen, Tao-Hsin Tung, Ching-Wen Chien

**Affiliations:** ^1^Institute for Hospital Management, Tsing Hua University, Shenzhen, China; ^2^Institute of Health Policy and Management, National Taiwan University, Taipei, Taiwan; ^3^Taiwan Association of Health Industry Management and Development, Taipei, Taiwan; ^4^Evidence-Based Medicine Center, Public Laboratory, Taizhou Hospital of Zhejiang Province Affiliated to Wenzhou Medical University, Linhai, China

**Keywords:** migraine, asthma, association, risk, systematic review, meta-analysis

## Abstract

**Objectives:** The purpose of this study was to determine the association between asthma and migraine and assess the risk for migraine in patients with asthma.

**Methods:** We systematically searched the Cochrane Library, PubMed, Medical Literature Analysis and Retrieval System Online (MEDLINE), and Excerpta Medica dataBASE (EMBASE) databases from inception to September 26, 2021, for indexed observational studies that examined either the odds or risk of migraine in subjects with asthma. The qualities of the included studies were evaluated using the Newcastle–Ottawa Scale. A random-effects meta-analysis was performed to calculate the odds ratio for case-control and cross-sectional studies and the risk ratio for cohort studies.

**Results:** Seven observational studies (four cross-sectional and three cohort studies) with a total of 549,534 study subjects were included in this systematic review and meta-analysis and selected for data extraction. Four articles were considered to be of moderate quality; other studies were considered to be of high quality. Asthma was associated with increased odds (OR, 1.85; 95%CI, 1.39–2.45) and risk of migraine (RR, 1.70; 95%CI, 1.52–1.90).

**Conclusions:** The available evidence that supports the existence of an association between asthma and migraine is limited. Clinicians should be aware that patients with asthma show both increased prevalence and incidence of migraine. Further studies are warranted to further clarify the relationship between asthma and migraine.

**Systematic Review Registration:** https://www.crd.york.ac.uk/PROSPERO/display_record.php?RecordID=185881, identifier: CRD42020185881.

## Introduction

Asthma is a chronic inflammatory disorder of the airways characterized by clusters of clinical respiratory symptoms. Many cells and cellular elements, such as mast cells, macrophages, and T lymphocytes, play a significant role in asthma (1). Reversible airway obstruction, airway inflammation, and airway hyperresponsiveness are the main features of asthma. Patients with asthma generally experience recurrent episodes of wheezing, coughing, dyspnea, and chest tightness, especially early in the morning and at night. Asthma is a serious global health problem affecting 1–18% of the population in different countries, estimates have indicated that 8% of adults aged 18 and over in the United States currently have asthma (2, 3). The global asthma prevalence is continuing to rise over the last decades (4). In addition, asthma is associated with many mental health disorders, such as major depressive disorder, bipolar disorder, and anxiety disorder (5).

Migraine is a severe neurobiological headache disorder characterized by various combinations of neurological, gastrointestinal, and autonomic changes (6). People with migraines may experience paroxysmal unilateral headache, vomiting, nausea, and severe sensitivity to light and sound, resulting in poor quality of life. Hours to days before a migraine attack, 20–60% of patients with migraine can experience premonitory symptoms, such as depression and cognitive dysfunction (7). Migraine is considered one of the most disabling medical diseases in the world. According to the findings of the Global Burden of Disease 2019, migraine ranked second among the global causes of disability, affecting an estimated 1.04 billion people worldwide (8).

Asthma is hypothesized to be an “acephalgic migraine” or a “pulmonary migraine” (9, 10). Previous studies have indicated that asthma and migraine share similar pathophysiology (11). Both asthma and migraine are characterized by inflammation and activation of smooth muscles in the airway or blood vessels. In addition, the shared environmental or genetic factors between asthma and migraine could induce subsequent diseases. Previous studies have demonstrated that long-term exposure to multiple air pollutants (such as air pollution, volatile organic compounds, oil, and gas wells, etc.) is a potential role for the occurrence or aggravation of asthma and migraine (12, 13). The polymorphisms of transient receptor potential receptors located on the trigeminal nerve and sensory afferents in the lungs are an example of genetic factors that account for the association between asthma and migraine (14). A previous systematic review and meta-analysis reported an association between asthma and migraine (15). However, the strength of the findings was limited by the lack of clear differentiation between migraines and general headaches. This study aimed to specifically explore the association between asthma and migraine while considering multiple confounders.

## Materials and Methods

### Literature Search

Two researchers (Chen and Kang) electronically searched the Cochrane Library, PubMed, Medical Literature Analysis and Retrieval System Online (MEDLINE), and Excerpta Medica dataBASE (EMBASE) databases for relevant studies published from inception to September 26, 2021, independently. The search string was “(asthma OR wheeze) AND (migraine OR hemicrania OR headache OR cephalgia OR cephalalgia)” with no limitations on languages. The search strategy for PubMed is shown in Table 1. The search strategy was modified for other databases. The reference lists of the review articles on the association between asthma and migraine were examined to identify articles not captured during the electronic search. The corresponding author of an article was contacted if the full text of the article was not available. This study was conducted according to the Meta-analysis of Observational Studies in Epidemiology (MOOSE) guidelines (16). The protocol of this systematic review was registered in PROSPERO under the number CRD42020185881.

**Table 1:** The search strategy of PubMed (similar search conducted in other databases).

**Database**	**Searching keywords**
PubMed	(1) asthma
	(2) wheeze
	(3) migraine
	(4) hemicrania
	(5) headache
	(6) cephalgia
	(7) cephalalgia
	(8) #1 or #2
	(9) #3 or #4 or #5 or #6 or #7
	(10) #8 and #9

### Study Selection

Observational studies were included if they fulfilled the following criteria: (i) cohort, case-control, or cross-sectional design; (ii) for cohort studies: an exposure group that consisted of people diagnosed with asthma and a control group that consisted of people without asthma; for case-control studies: the outcome reported were migraine, the case group consisted of people diagnosed with asthma and the controls are healthy people without asthma; for cross-sectional studies: the cases reported were migraine in a selected population and the exposure of interest is asthma; (iii) study outcome was either the odds or risk of migraine in subjects with and without asthma; and (iv) in the English language. Studies inclusive of individuals with a current and/or history of asthma or migraine were included. Studies were excluded if they were randomized controlled trial design, comments, editorials, conference abstracts, reviews, meta-analysis, or animal studies. It has been confirmed that childhood asthma and adult asthma are not exactly the same in pathogenesis (17), and migraines in children can manifest in ways that are markedly different from adult migraines (18). Hence, we excluded asthma and migraine before adolescence (<12 years) to minimize heterogeneity concerning age. In addition, we excluded case series without a control group or observational studies that did not provide enough data on the odds or risk of migraine in subjects. We scanned the titles and abstracts in the search results and obtained the full texts of potentially eligible studies to determine whether they met the predetermined selection criteria. The studies were independently selected by two authors (Chen and Kang). Disagreements were resolved through consensus.

### Data Extraction and Quality Assessment

The following data were extracted from all the included studies using a data extraction form: first author, publication year, country, study design, study subjects, ascertainment of asthma and migraine, and results. We categorized the included studies into two types: (i) cross-sectional or case-control studies containing an asthma group and a non-asthma group, and (ii) cohort studies containing an asthma group and a non-asthma group. The quality of the case-control and cohort studies were assessed according to the Newcastle–Ottawa Scale (NOS) (19), while the cross-sectional studies were evaluated using a modified NOS (20). The NOS includes the following three domains that assess the quality of observational studies: selection of study groups (four items), comparability (one item), and exposure assessment for the case-control studies or outcome assessment for the cohort studies (three items). The details of each domain differ for the assessment of case-control studies and cohort studies. A study can be awarded one star for each item in the selection and exposure/outcome domains and two stars in the comparability domain. A study is considered high-quality research if it is awarded 7 or more stars, moderate-quality if it is awarded between 2 and 7 stars, and poor-quality if it is awarded ≤2 stars (16). The modified NOS evaluates seven methodological items, a study is considered high-quality research if it is awarded 6 or more stars, moderate-quality if it is awarded between 2 and 6 stars, and poor-quality if it is awarded ≤2 stars (20). The details and assessment of the included studies were abstracted by one author and reviewed by another author for accuracy. Discrepancies were resolved through consensus.

### Statistical Analysis

The Review Manager 5.3 software (Nordic Cochrane Centre, Copenhagen, Denmark) was used to conduct this meta-analysis. The risk ratios (RRs) or odds ratios (ORs) with 95% CIs were aggregated for the cohort studies to report the final pooled estimation, whereas the ORs were calculated for the case-control/cross-sectional studies. The heterogeneity was evaluated by using the *I*^2^ statistic and *Q*- statistic (21). The *P*_*Q*_ < 0.10 was considered indicative of statistically significant heterogeneity. The *I*^2^ statistic was used to establish the percentage of the total variation. An *I*^2^-value of ≥50% represents substantial heterogeneity. We performed a random-effects model meta-analysis if the *I*^2^*-*value was ≥50%; otherwise, a fixed-effects model was used.

The overall link between asthma and migraine was assessed for the primary analysis. Subgroup analyses were also conducted according to the (i) ages (adults ≥18 years and adolescents between age 12 and 18 years), (ii) methodological quality (moderate and high), and (iii) adjusted for factors (comorbid disorders and medication use).

## Results

### Characteristics of the Included Studies

**Figure 1:**
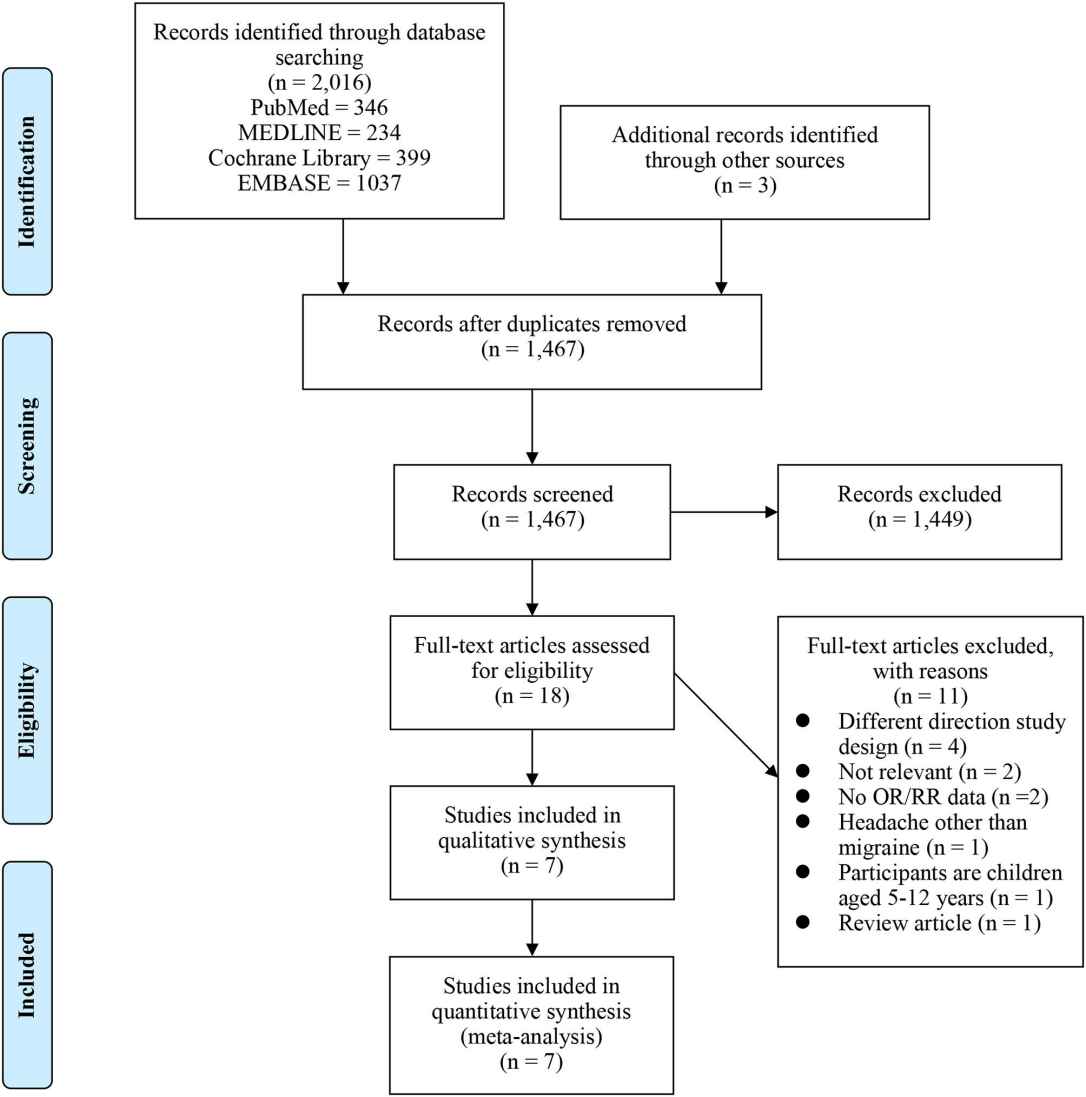
Preferred Reporting Items for Systematic Reviews and Meta-Analyses (PRISMA) study flow chart.

As illustrated in Figure 1, 1,467 articles were identified in the search after the duplicates were removed. Finally, 18 full texts were assessed for eligibility among which seven studies (three cohort studies and four cross-sectional studies) were included in the present systematic review and meta-analysis (11, 14, 22–26). Supplementary Table S1 provided the excluded studies with the reasons for exclusion after the assessment of the full text.

The characteristics of the seven included studies are summarized in Table 2. These studies were published between 2007 and 2019, with a total of 549,534 study subjects. Of these, three cohort studies were conducted in Taiwan, Korea, and the United States, while four cross-sectional studies were conducted in Spain, Norway, Israel, and Sweden. The age of the participants was older than 12 years in all included studies. Among the three cohort studies, the duration of the follow-up for migraine was more than 10 years in two studies (11, 22) but only 1 year in the other study (14). For the diagnosis or symptoms of migraine, three studies used self-report diagnosis or answers from questionnaires (23, 24, 26) and two studies used the International Classification of Diseases (ICD) code for migraine criteria (11, 22). A study conducted by Martin et al. investigated the risk of developing chronic migraine to episodic migraine among individuals with or without asthma (14). The participants completed a written headache questionnaire and made a diagnosis of migraine according to the International Classification of Headache Disorder-II (ICHD-II) criteria. One study made the diagnosis of migraine by neurologic specialists who reviewed the data from a medical evaluation carried out by family physicians (25).

**Table 2:** Characteristics of included studies.

**Study**	**Study design**	**Target group**	**Case/exposed group**	**Control group**	**Ascertainment of asthma**	**Ascertainment of migraine**	**Results**
Peng et al. (22), Taiwan	Cohort study	Adults (> 12 years)	25,560 with asthma (12,183 males and 13,377 females)	102,238 subjects matched by sex, age (every 5 years span), and index year (48,730 males and 53,508 females)	ICD-9-CM 493	ICD-9-CM-346	HR 1.45 (95% CI 1.33–1.59) adjusted for asthma, sex, age, CCI score, medication, and annual OPD visits
Kim et al. (11), Korea	Cohort study	Adults (>20 years)	113,059 with asthma (42,172 males and 70,887 females)	113,059 subjects matched by age, sex, income, and region of residence (42,172 males and 70,887 females)	• Asthma: ICD-10: J45; • Status asthmaticus: ICD-10: J46	• Migraine with aura: ICD-10: G43; • Migraine without aura: ICD-10: G431	HR 1.47 (95%CI 1.41–1.53) adjusted for depression history, and CCI calculated without pulmonary disease
Martin et al. (14), US	Cohort study	Adults (≥18 years)	746 with asthma and episodic migraine (118 males and 628 females)	3,700 respondents met criteria for episodic migraine (737 males and 2,963 females)	Six-item asthma questionnaire from ECRHS-II	ICHD-II criteria	OR 2.01 (95% CI 1.24-3.26) adjusted for age, sex, and income, current preventive medication use, monthly headache day frequency, medication overuse
Fernández-de-Las-Peñas et al. (24), Spain	Cross-sectional study	Adults (≥16 years)	1,683 with asthma	27,795 individuals from the general Spanish population	Self-reported diagnosis by questionnaire	Self-reported diagnosis by questionnaire	OR 1.62 (95% CI 1.27–2.05) adjusted for sex, age, monthly income, self-rated health, sleep habits, and concomitant chronic diseases
Aamodt et al. (23), Norway	Cross-sectional study	Adults (≥20 years)	2,511 with asthma (1,120 males and 1381 females)	42,472 individuals from the general population (19,452 males and 23,020 females)	Having had asthma at one time or another and attacks of wheezing or breathlessness during the last 12 months	Headache attacks lasting 4–72 h with at least 1 characteristics (pulsating quality, unilateral location, and aggravation by physical activity) and at least 1 symptoms (nausea, photophobia, and/or phonophobia) during headache	OR 1.51 (95% CI 1.3–1.7) adjusted for sex, age, education level, and smoking (pack years)
Graif et al. (25), Israel	Cross-sectional study	Adolescents (17 years)	4,581 with asthma	109,090 military candidates	The specialists in pulmonology made the diagnosis	The specialists in neurology made the diagnosis	OR 1.42 (95% CI 1.19–1.68) adjusted for allergic rhinitis, gender, residence, origin, no. of siblings, and BMI
Tsiakiris et al. (26), Sweden	Cross-sectional study	Adults (18–79 years)	164 with asthma	2,876 individuals without asthma	Self-reported diagnosis by questionnaire	Self-reported diagnosis by questionnaire	OR 1.87 (95% CI 1.01–3.48) adjusted for sex and education

The definition used for asthma was variable between studies. Martin et al. defined the presence and severity of asthma based on a self-reported questionnaire in the European Community Respiratory Heath Survey-II (ECRHS-II) (14). Peng et al. compared newly diagnosed asthma patients (ICD-9-CM 493) who received treatment involving inhaled corticosteroids (ICS), systemic corticosteroids, or inhaled beta-2 agonists against a non-asthma cohort matched according to sex, age, and index year (22). One study selected participants who were diagnosed with asthma (ICD-10: J45) or status asthmaticus (ICD-10: J46) by physicians more than two times and the asthma-related medications histories, including ICSs, ICSs combined with long-acting β2-agonists (LABAs), short-acting β2-agonists (SABAs), systemic LABAs, oral leukotriene antagonists (LTARs), xanthine derivatives, or systemic corticosteroids; and the control group consist of participants without asthma and were matched 1:1 by age, group, sex, income group, region of residence, and past medical history (hypertension, diabetes, and dyslipidemia) with the participants with asthma (11). Three cross-sectional studies made the diagnosis of asthma by self-report diagnosis or answers from questionnaires (23, 24, 26). One study made the diagnosis of asthma by pulmonary specialists who made the assessment and classification based on documentation from the family physician of the subject, personal medical interview, and physical examination (25).

### The Methodological Quality of the Included Studies

The methodological quality of the included studies was assessed based on the NOS and the modified version of the NOS. As presented in Supplementary Table S2, three studies were considered as high-quality (11, 22, 25). Three cross-sectional studies (23, 24, 26) were awarded four stars and appraised of moderate-quality because they ascertained the exposure and outcome by self-reported and the comparability between respondents and non-respondents was unsatisfactory. One cohort study (14) was awarded six stars and considered as moderate-quality because the exposed cohort was selected from the episodic migraine group and the ascertainment of exposure and outcome was by self-report questionnaire.

### Risk of Migraine in Patients With Asthma in Cohort Studies

**Figure 2:**
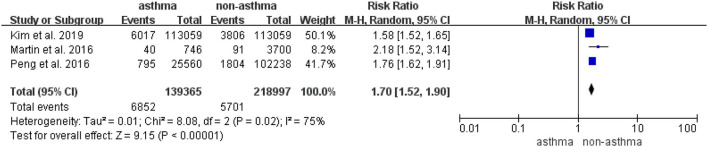
Risk of migraine in patients with asthma in the cohort studies.

Three cohort studies with a total of 139,365 patients with asthma and 218,997 controls provided data for the analysis of the risk of migraine in patients with asthma (11, 14, 22). As illustrated in Figure 2, substantial heterogeneity was found (*I*^2^ = 75%, *P*_Q_ < 0.10). The RR of migraine for the patients with asthma compared with the controls ranged from 1.58 (95%CI, 1.52–1.65) to 2.18 (95%CI, 1.52–3.14). In the pooled estimates, the total RR was 1.70 (95%CI, 1.52–1.90), indicating that patients with asthma had a significantly increased risk for migraines.

### Odds of Migraine in Patients With Asthma in Case-Control and Cross-Sectional Studies

**Figure 3:**
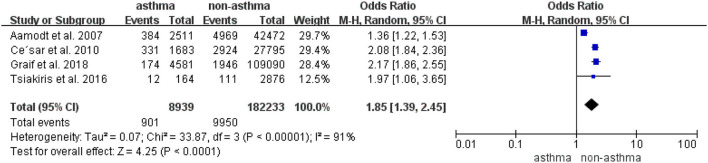
Odds of migraine in patients with asthma in the case-control and cross-sectional studies.

Four cross-sectional studies with 8,939 patients with asthma and 182,233 controls provided data for the analysis of the odds of migraine in patients with asthma (23–26). As shown in Figure 3, in this random-effects meta-analysis of four cross-sectional studies, asthma was associated with an increased risk of migraine (pooled OR, 1.85; 95%CI, 1.39**–**2.45), and substantial heterogeneity was detected across the studies (*I*^2^ = 91%, *P*_*Q*_ < 0.10).

### Subgroup Analysis

The results from the subgroup meta-analysis according to several factors are summarized in Table 3. Asthma was consistently associated with migraines in the subgroup meta-analysis according to age, methodological quality, and adjustment for comorbid disorders and medication use. With respect to age, the association between asthma and migraine in the subgroup of subjects aged 18 and older was lower than the whole group consisting of all ages. As for the methodological quality, the link between asthma and migraine was almost the same in different methodological qualities. In addition, the association between asthma and migraine was reduced after adjusting the factors of comorbid disorders and medication use.

**Table 3:** Association between asthma and migraine in subgroup meta-analysis by various factors.

**Factors**	**Number of studies**	**Summary OR (95% CI)**	***P* for heterogeneity**	**Heterogeneity, *I*^2^ (%)**
All	7	1.80 (1.59–2.03)	<0.001	86%
**Ages**				
All ages (≥12 years)	7	1.80 (1.59–2.03)	<0.001	86%
≥18 years	4	1.60 (1.36–1.87)	<0.001	7%
<18 years	1	2.17 (1.86–2.55)	–	–
Methodological quality				
High	3	1.81 (1.57–2.08)	<0.001	87%
Moderate	4	1.83 (1.35–2.50)	<0.001	89%
Adjusted for following factors				
Comorbid disorders^*^	4	1.47 (1.42–1.52)	0.84	0%
Medication use^**^	2	1.56 (1.19–2.04)	0.19	41%

* *Comorbid disorders include chronic pulmonary disease, cerebrovascular disease, diabetes mellitus, allergic rhinitis, and other chronic diseases*.

** *Medication use include both asthma medication and migraine medication*.

## Discussion

To our knowledge, our study is not the first meta-analysis to examine the association between asthma and migraine. The previous meta-analysis by Sayyah et al. (15) indicated the bidirectional association between asthma and migraine. But they mistakenly considered headache the same as migraine and included studies both on headache and migraine in the meta-analysis, which may reduce the accuracy and validity of the outcome. In addition, both children and adults with asthma were included in their meta-analysis without an appropriate acknowledgment that the pathogenesis and manifestations may differ between the age groups. In addition, the comorbidities and medication use in asthma or migraine may account for their relationship, but this systemic review and meta-analysis did not mention that. Whereas, our study made a clear distinction between migraine and headache, and only included adults for the meta-analysis, which may increase the quality of evidence for the outcome. In addition, we added more recent studies (two cross-sectional studies and one cohort study) and analyzed the data separately according to study design and multiple confounders, thus providing more rationality evidence on the association between asthma and migraine than previous studies.

Our study showed that asthma was associated with a moderately increased risk of migraines. Similar results were found in the subgroup meta-analyses according to age, methodological quality, and adjustment for confounding factors. When analyzing only cohort studies, this association remained significant, suggesting asthma as a potential risk factor for migraines. Nevertheless, owing to the limited number and quality of cohort studies included in the meta-analysis, the general conclusion is far from definitive at this stage.

The mechanisms underlying the association between asthma and migraine could be thought of from the perspective of inflammation, immune dysfunction, and environmental triggers. Atopy, parasympathetic hyperactivity, and elevated neuropeptide release are the main pathophysiological factors of inflammation and immune dysfunction that play a crucial role in the pathogenesis of asthma and migraine. For patients with asthma, exposure to allergens, such as grass, pollen, and animal hair, may trigger an airway hypersensitivity response mediated by mast cells. Plasma cells produce allergen-specific immunoglobulin E antibodies due to the hypersensitivity response, which can stimulate mast cells to release a variety of inflammatory mediators (e.g., histamine, prostaglandins, chymase, tryptase, etc.) and cytokines (TGFβ1, TSLP, IL-33, IL-23, etc.) (27, 28). In migraines, meningeal mast cells are in proximity to meningeal blood vessels and nociceptive axons. When meningeal mast cells are activated by allergens, they degranulate and secrete large numbers of inflammatory cytokines [e.g., Tumor necrosis factor (TNF)], leading to neurogenic inflammation, which activates or sensitizes nociceptors and neurons and influences the reactivity of signal nociceptors to the brain, thereby triggering the genesis of migraine (29). A previous study has shown that allergy shots can reduce the frequency of migraine by up to 48% in young patients with atopic migraine; a finding which strongly supports that atopy is a potential mechanism of migraine (30). In addition, a previous study had shown that exposure to tobacco smoke appeared to skew immune responses mainly by altering the immune functions of varieties of immune cells and aggravating allergic inflammation and sensitization, which is implicated in the pathogenesis of asthma and migraine (31, 32).

Asthma and migraine are characterized by parasympathetic hyperactivity. Parasympathetic nerves innervate the lungs and control the symptoms of asthma through peripheral muscarinic receptor signaling. Activated by inflammatory mediators, the parasympathetic nerves signal the airway smooth muscles and secretory glands, mediating airway stenosis and increased mucus secretion, which are the key features of an asthmatic response (33). Similarly, the parasympathetic nerves supply meningeal vessels via the sphenopalatine ganglion. Many neurotransmitters, such as acetylcholine, vasoactive intestinal polypeptide, and pituitary adenylate cyclase-activating polypeptide, are released from parasympathetic nerves, which play important roles in the mechanism of migraine (34). A previous study demonstrated that the blood velocity in the middle cerebral artery slowed during a migraine attack, reflecting the vasodilation of cerebral vessels, which is a result of increased parasympathetic tone (35). Thus, we hypothesized that the generalized parasympathetic hyperactivity observed in patients with asthma might contribute to their increased susceptibility to migraines.

Both asthma and migraine are also characterized by elevated neuropeptide release. Activated by endogenous or inhaled irritants, the airway C-fiber sensory nerves release various neuropeptides, such as calcitonin gene-related peptide, neurokinins, and tachykinins, inducing mucus secretion, inflammation, and constriction and hyperactivity of the airway, all of which are key features in the pathophysiology of asthma (36). Similarly, neuropeptides may initiate neurogenic inflammation and participate in the sensitization of the trigeminal system, both of which play key roles in the initiation of migraines.

It is also possible that migraines are not directly caused by asthma; shared environmental triggers may be responsible for the association between them. Studies have shown that air pollution, such as high concentrations of PM2.5, PM10, and NO_2_, can induce asthma and even aggravate its symptoms (37). Similarly, the rate of emergency treatments for migraines increases due to exposure to air pollution (38). Considering that asthma is diagnosed earlier than migraine, we hypothesized that the impact of environmental triggers on asthma precede their impact on migraine. Psychological factors also account for the association between asthma and migraine. The findings of previous studies suggest that asthma and mental health disorders share genetic risk factors (5). Asthma is associated with a 1.81-fold increased risk for mental health disorders (39). Depression and stressful events are the main risk factors for migraine or the progression of acute migraine to chronic migraine (40). Thus, effective control of environmental and psychological factors may reduce the occurrence of asthma and migraine.

Additionally, sleep-related disorders could be a latent confounder account for the relationship between the two diseases. In asthmatic patients, poor quality sleep and sleep disturbance are highly prevalent, the symptoms of increased sleep latency, a reduction in slow-wave sleep, and reduced sleep efficiency can be seen in asthmatic patients and have been objectively demonstrated in the previous studies (41). In migraines, poor sleep has been consistently reported both in precipitating and during attacks and physicians regularly consider sleep as a treatment for migraine (such as selecting medications with sedative sides effects) (42, 43). Many studies have reported that the prevalence of migraine is higher in patients with sleep disorders (such as insomnia, parasomnias, sleep-related movement disorders) compared with those without (44).

The medication use in asthmatic patients may account for the relationship between asthma and migraine. Theβ_2_-adrenoceptor agonists (such as salbutamol, salmeterol) are the most effective and widely used drugs that rapidly reverse bronchoconstriction and have become standard bronchodilators for the emergency room treatment of asthma, and as a day-to-day reliever medication (45). A study conducted in a United Kingdom cohort of 15,407 patients found that headache was associated with the use of salmeterol and was one of the most common adverse reactions to salmeterol (46). Hence, we hypothesized that the series of drugs to treat asthma may be a risk factor for migraines. Moreover, β-blockers are the most widely used class of drugs in prophylactic migraine treatment (47). However, previous studies indicated that the administration of β-blockers could increase airway hyperresponsiveness and airflow limitation which could induce asthma symptoms or exacerbations in people with asthma (48). Therefore, the lack of administration of β-blockers in asthmatic patients may aggravate the symptoms of migraine. In addition, asthma is often associated with numerous comorbidities which may mediate its relationship with migraine. A nationwide case-control study (*N* = 40,477,745) conducted in Germany found that more than 50% of patients with asthma had mental diseases and every fourth patient had a diagnosis of depressive episode and every fifth patient had sleep disorders (49). And these diseases have been confirmed to have a link with migraines, historically (44, 50, 51). Our subgroup meta-analysis according to the adjustment of medication use and comorbidities found that the association between asthma and migraine was lower than the whole group meta-analysis, which could provide evidence for our hypothesis.

### Limitations

The causal relationship between asthma and migraine was difficult to explore in this study because only three cohort studies were included in the analysis. In addition, the relative statistical power of the results would be reduced owing to the small sample size of the study. Further studies are needed to identify whether asthma is an independent risk factor for migraines. Moreover, due to the limited information in the included studies, we could not conduct subgroup analyses based on sex, region, disease severity, or asthma phenotypes. Additionally, the association between asthma and different types of migraines was not analyzed. Furthermore, we did not include asthma and migraine before adolescence to decrease the heterogeneity of the data. Further studies should include both children and adults in the analysis.

## Conclusions

The findings of this study suggest that a relationship exists between asthma and migraine. Clinicians should be aware that patients with asthma show an increased incidence of migraines. Although asthma and migraine may have similar pathogeneses, it is difficult to determine whether there is a direct relationship between them. More large-scale prospective studies are warranted to verify the findings of this study and to provide more information on the association between asthma and migraine.

## Data Availability Statement

The original contributions presented in the study are included in the article/Supplementary Material, further inquiries can be directed to the corresponding author/s.

## Author Contributions

L-LK made great contributions to data acquisition, analysis and interpretation, and manuscript writing. P-EC has done lots of work on data acquisition and manuscript revision. T-HT and C-WC made substantial contributions to the design and conception of the study and revised the manuscript. All authors have read and approved the final manuscript.

## Conflict of Interest

The authors declare that the research was conducted in the absence of any commercial or financial relationships that could be construed as a potential conflict of interest.

## Publisher's Note

All claims expressed in this article are solely those of the authors and do not necessarily represent those of their affiliated organizations, or those of the publisher, the editors and the reviewers. Any product that may be evaluated in this article, or claim that may be made by its manufacturer, is not guaranteed or endorsed by the publisher.

## Abbreviations

MOOSE: Meta-analysis of Observational Studies in Epidemiology
RCT: randomized controlled trial
NOS: Newcastle-Ottawa Scale
RR: risk ratio
OR: odds ratio
CI: confidence interval
ICD: International Classification of Diseases
ICHD-II: International Classification of Headache Disorder-II
ECRHS-II: European Community Respiratory Health Survey-II
ICS: inhaled corticosteroid
LABA: long-acting β2-agonist
SABA: short-acting β2-agonist
LTAR: oral leukotriene antagonist
NO_2_: nitrogen dioxide.
