# Supplementary material for: Association Between Asthma and Migraine: A Systematic Review and Meta-Analysis of Observational Studies
Source: Front Allergy. 2021 Dec 1;2:741135. doi: 10.3389/falgy.2021.741135 (PMC8974722; doi:10.3389/falgy.2021.741135)
Supplement: Supplementary file 1 [file Table_1.DOCX]

**Supplemental material**

**Supplemental Table 1. Excluded studies with reasons**

| Most of the excluded studies violated more than one eligibility criterion. Only one of the violated criteria is narrated for each study. Among 9 excluded studies: 4 studies had the different direction study design, they reported asthma not migraine as an outcome assessment (1-4), 1 study is a systematic review study rather than an observational study (5), 2 studies are irrelevant topics (1 study reported the relationship between the risk of asthma in children and the mother’s propensity to migraine and 1 study reported asthma phenotypes and associated comorbidities) (6,7), 2 studies didn’t report the odds or risk of migraine in subjects with and without asthma (8,9), in 1 study participants are children aged 5-12 years (10), in 1 study patients with daily headache (not with migraine diagnosis specifically) (11). |
| --- |
| 1. Peng YH, Chen KF, Liao WC, et al. Association of migraine with asthma risk: A retrospective population-based cohort study. Clin Respir J. 2018;12(3):1030-1037. 2. Chen YC, Tang CH, Ng K, et al. Comorbidity profiles of chronic migraine sufferers in a national database in Taiwan. J Headache Pain. 2012;13(4):311-9. 3. Becker C, Brobert GP, Almqvist PM, et al. The risk of newly diagnosed asthma in migraineurs with or without previous triptan prescriptions. Headache. 2008;48(4):606-10. 4. Davey G, Sedgwick P, Maier W, et al. Association between migraine and asthma: matched case-control study. Br J Gen Pract. 2002;52(482):723-7. |
| 1. Sayyah M, Saki-Malehi A, Javanmardi F, et al. Which came first, the risk of migraine or the risk of asthma? A systematic review. Neurol Neurochir Pol. 2018;52(5):562-569. |
| 1. Chen TC, Leviton A. Asthma and eczema in children born to women with migraine. Arch Neurol. 1990;47(11):1227-30. 2. Machluf Y, Farkash R, Rotkopf R, et al. Asthma phenotypes and associated comorbidities in a large cohort of adolescents in Israel. J Asthma. 2020;57(7):722-735. |
| 1. Czerwinski S, Gollero J, Qiu C, et al. Migraine-asthma comorbidity and risk of hypertensive disorders of pregnancy. J Pregnancy. 2012; 2012:858097. 2. Dirican N, Demirci S, Cakir M. The relationship between migraine headache and asthma features. Acta Neurol Belg. 2017;117(2):531-536. |
| 1. Wilkinson IA, Halliday JA, Henry RL, et al. Headache and asthma. J Paediatr Child Health. 1994;30(3):253-6. |
| 1. Lee YS, Lee GD, Lee JS, et al. Is daily headache related to asthma? Results from a population-based survey. J Asthma. 2013;50(7):745-50. |

**Supplemental Table 2. Quality evaluation according to the Newcastle-Ottawa Scale (NOS) and modified NOS**

| **Study** | **Selection** | **Comparability** | **Exposure/outcome** | **NOS score** | **Quality** |
| --- | --- | --- | --- | --- | --- |
| Peng et al., 2016 | **** | ** | *** | 9/9 | High |
| Kim et al., 2019 | **** | ** | *** | 9/9 | High |
| Martin et al., 2016 | ** | ** | ** | 6/9 | Moderate |
| Ce´sar et al., 2010 | * | ** | * | 4/7 | Moderate |
| Aamodt et al., 2007 | * | ** | * | 4/7 | Moderate |
| Graif et al., 2018 | ** | ** | ** | 6/7 | High |
| Tsiakiris et al., 2016 | * | ** | * | 4/7 | Moderate |

The number of * corresponds to the number of items assessed positively in each category
